# Books Reviewed

**Published:** 1879-03

**Authors:** 


### Books Reviewed.

*Principles and Practice of Surgery.* By JOHN ASHURST, M. D., Professor of Clinical Surgery in the University of Pennsylvania; Sugeon to the Episcopal Hospital and the Children's Hospital; Consulting Surgeon to St. Christopher's Hospital of the Good Shepherd. *Second edition*, enlarged and thoroughed revised. With five hundred and forty-two illustrations. Philadelphia: Henry C. Lee. 1878.

This work furnishes in a condensed and concise manner description of the present most approved methods of surgical practice. The whole subject matter of surgery is presented in its pages in form to exactly meet the requirements of general practitioners who are engaged in both medical and surgical practice. It is a book of ready reference for general use among practitioners, and at the same time a standard authority of great value. The student in medicine and surgery will find it the most satisfactory text-book on all surgical subjects, and to him it is of special value, having been brought up to contain and explain all recent observations and improvements in surgical science.

The arrangement of this volume is similar to the first edition, though it has been carefully revised and much new material added. In its present revised and illustrated edition it fully meets the requirements of practitioners generally, while medical students everywhere will appreciate its completeness and exact adaptation to their wants. Space prevents expression of our full estimate of its merits. We can only briefly indicate its general merits, and advise physicians and students that it is concise, includes the whole field, is beautifully illustrated, and in all respects merits first place as surgical text-book.

*The American Quarterly Microscopical Journal.* Edited by ROMYN HITCHCOCK, and published by Hitchcock and Walls, 150 Nassau Street, New York.

The first number of this work is before us, and we consider it well calculated to fill a long felt vacancy among the Microscopic workers of America. Similar Journals have been in existence in Europe for many years, and have been looked for and perused with great eagerness on this side the Atlantic. We are pleased therefore to see so good a specimen of American enterprise It bids fair to be at least equal to those of a similar character on the other side. The paper and typography are excellent, and the illustrations are both numerous and finely executed.

The contributors are among the most distinguished microscopic scientists of the old and new world.

We notice an article on the Sting of the Honey Bee (*Apis Millifica*) by Prof. J. D. Hyatt, and an article by Prof. H. L. Smith of Hobart College, on New Diatoms, some of them found in our own Niagara water. Many other valuable contributions fill its pages, to which is appended Reports of the Microscopic Convention, held at Indianapolis last August. Also the Transactions of the Microscopal Society of New York.

From a list of forthcoming articles, we notice one by Prof. I. N. Danforth, entitled "A Typical case of Tubercular Meningitis," with steel plates. Also one on "The Microscopic Character of Natural, and Artificial Butter," by the Editor; and "Observations on the Structure of Ophioglossum" (with plate) by Prof. Mark Harrington.

———:o:———

